# Supplementary material for: Transcriptome analysis unravels the biocontrol mechanism of Serratia plymuthica A30 against potato soft rot caused by Dickeya solani
Source: PLoS One. 2024 Sep 6;19(9):e0308744. doi: 10.1371/journal.pone.0308744 (PMC11379202; doi:10.1371/journal.pone.0308744)
Supplement: Attachment — Submitted filename: Comments on PLOS ONE paper.docx [file pone.0308744.s005.docx]

Hadizadeh et al. manuscript entitled “Transcriptome analysis unravels the biocontrol mechanism of *Serratia plymuthica* A30 against potato soft rot caused by *Dickeya solani*” looks very interesting and technically sound. This type of study should be recommended for publication. However, there are some points requiring consideration.

1. The Abstract should reflect the problem, the method, and the conclusions, please further improve
2. In the last paragraph of the Introduction, please state your motivation, goals and objectives, the novelty of this research, and potential contribution to the literature.
3. Make changes in Keywords; because most of the words are included in the paper title.
4. In page 6, on lines 203-206, mention the software and the corresponding parameters used for preprocessing of the sequenced data.
5. In page 6, on line 218, mention the version of NOISeq used for the analysis.
6. In page 7, on line 250, write “ p ” instead of “ P ”. p-value should always be written as “p” or “p-value”. Correct it throughout the manuscript.
7. All the figures in the manuscript are of very poor quality making it unable to read the labels. They should be replaced with the high-resolution images.
8. Authors effort to illustrate the role of plant hormones SA, JA, ethylene, ABA, Auxin, cytokinin and GA is impressive. Figure 8 illustration on proposed defense response mechanism of potato tuber towards *S. plymuthica* A30 and infection by *D. solani* clearly portrayed the complex responses and interplay of several plant hormones. It would be better to include the same in the manuscript under separate section with a brief discussion.
9. Conclusion can be improved further.
10. Check the manuscript for all the typo errors and correct accordingly.
